# Biohybrid elastin-like venous valve with potential for *in situ* tissue engineering

**DOI:** 10.3389/fbioe.2022.988533

**Published:** 2022-09-21

**Authors:** Fernando González-Pérez, Sergio Acosta, Stephan Rütten, Caroline Emonts, Alexander Kopp, Heinz-Werner Henke, Philipp Bruners, Thomas Gries, J. Carlos Rodríguez-Cabello, Stefan Jockenhoevel, Alicia Fernández-Colino

**Affiliations:** ^1^ Bioforge Lab (Group for Advanced Materials and Nanobiotechnology), CIBER-BBN, Edificio LUCIA, Universidad de Valladolid, Valladolid, Spain; ^2^ Department of Biohybrid and Medical Textiles (BioTex), AME–Institute of Applied Medical Engineering, Helmholtz Institute, RWTH Aachen University, Aachen, Germany; ^3^ Electron Microscopy Facility, Uniklinik RWTH Aachen, Aachen, Germany; ^4^ Institut für Textiltechnik Aachen (ITA), RWTH Aachen University, Aachen, Germany; ^5^ Meotec GmbH, Aachen, Germany; ^6^ Innovative Tomography Products GmbH, Bochum, Germany; ^7^ Klinik für Diagnostische and Interventionelle Radiologie, Universitätsklinikum Aachen, Aachen, Germany; ^8^ AMIBM-Aachen-Maastricht-Institute for Biobased Materials, Maastricht University, Maastricht, Netherlands

**Keywords:** miniaturized valve, cell-free, bioabsorbable, elastin-like recombinamer, magnesium stent

## Abstract

Chronic venous insufficiency (CVI) is a leading vascular disease whose clinical manifestations include varicose veins, edemas, venous ulcers, and venous hypertension, among others. Therapies targeting this medical issue are scarce, and so far, no single venous valve prosthesis is clinically available. Herein, we have designed a bi-leaflet transcatheter venous valve that consists of (i) elastin-like recombinamers, (ii) a textile mesh reinforcement, and (iii) a bioabsorbable magnesium stent structure. Mechanical characterization of the resulting biohybrid elastin-like venous valves (EVV) showed an anisotropic behavior equivalent to the native bovine saphenous vein valves and mechanical strength suitable for vascular implantation. The EVV also featured minimal hemolysis and platelet adhesion, besides actively supporting endothelialization *in vitro*, thus setting the basis for its application as an *in situ* tissue engineering implant. In addition, the hydrodynamic testing in a pulsatile bioreactor demonstrated excellent hemodynamic valve performance, with minimal regurgitation (<10%) and pressure drop (<5 mmHg). No stagnation points were detected and an *in vitro* simulated transcatheter delivery showed the ability of the venous valve to withstand the implantation procedure. These results present a promising concept of a biohybrid transcatheter venous valve as an off-the-shelf implant, with great potential to provide clinical solutions for CVI treatment.

## Introduction

Lower extremity chronic venous insufficiency (CVI) represents a global health issue worldwide, affecting between 5 and 33% of the adult population (Ruckley, 1997; Mervis et al., 2019). The clinical manifestations range from cosmetic issues to severe complications such as venous ulcers, thrombus formation, painful swelling, edema, and distal venous hypertension (Raju, 1983; Brand et al., 1988; Raju et al., 2000; Cesarone et al., 2002; Eberhardt and Raffetto, 2005; Fernández-Colino and Jockenhoevel, 2021). Classic therapies targeting CVI include anticoagulants and compression devices, but such strategies target just the symptoms and do not provide a functioning valve.

In order to provide a functioning valve, one option consists of performing autologous transplantation, i.e. a vein segment that contains a functional valve is removed and implanted into another location. However, this approach presents several disadvantages such as high patient comorbidity and limited availability. As an alternative, allo- and xenograft concepts have also been explored and even reached clinical trials (Dalsing et al., 1999; Neglén and Raju, 2003; Gale et al., 2004). However, despite their broader availability, their performance was proven suboptimal, with high failure rates due to thrombosis and immunogenic rejection (Neglén and Raju, 2003; Pavcnik et al., 2008). To overcome these limitations, venous valves based on polymeric and metallic materials appeared as a promising solution. For example, venous valves made of pellethane, platinum, pyrolytic carbon-covered titanium, polyether urethane, polytetrafluoroethylene, and poly (vinyl-alcohol) have been reported with their respective *in vivo* studies. However, these concepts failed to meet clinical standards, with occlusion, hyperplasia, fibrosis, and thrombosis among the main drawbacks observed (Hill et al., 1985; Taheri et al., 1988; Uflacker, 1993; Taheri and Schultz, 1995; Midha, 2009; Jan de Borst and Moll, 2012; Boersma et al., 2017).

The aforementioned issues of previous concepts motivated the development of venous valves based on biodegradable polymers, which combined with the principles of tissue engineering (TE) could lead to the development of native valve replacements. In this regard, Weber et al. described a valve made of polyglycolic acid (PGA) with poly-4-hydroxybutyrate (P4HB) combined with a Nitinol stent, which was cultured with ovine mesenchymal stem cells (MSCs) and ovine endothelial cells (ECs) *in vitro*. However, significant endothelial cell loss was observed during the crimping procedure (Weber et al., 2013). In a different study, Syedain et al. used fibrinogen cultured with ovine fibroblasts to create a collagenous matrix, which was attached to a Nitinol stent and further decellularized. Its hydrodynamic performance met the requirements for a functional venous valve, and host cell endothelialization was reported *in vivo* in an ovine model (Syedain et al., 2019). However, the valve experienced a fusion of the leaflets and clot formation after 8 weeks post-implantation. Additionally, while the valve was intended as an *in situ* tissue-engineered implant, the concept relied on a decellularized matrix, and the production process involved cell culture and *in vitro* conditioning. Therefore, this concept did not fully exploit the principles of an *in situ* “culture free” TE approach.

An *in situ* “culture free” TE concept benefits from off-the-shelf availability as an implant, lower regulatory burden for clinical translation and no need for tissue harvest for cell isolation. Indeed, cardiovascular TE strategies are increasingly moving from a classical to an *in situ* approach, in which the implants are subjected to endogenous regeneration, and progressively transformed into autologous substitutes. However, this strategy is highly demanding regarding the implant’s biofabrication material. Specifically, the cell-free implant has (i) to take over the functionality without the help of any pre-conditioning *in vitro* phase, and (ii) to provide a microenvironment suitable for cell infiltration and tissue generation *in situ* (Billiet et al., 2012).

One of the key functional components of native vascular tissues is elastin-rich structures, which play crucial mechanical and bioactive roles (Wang et al., 2021). The lack of elastin in a vascular device contributes to stenotic and thrombotic events, thus leading to vascular implant failure. Elastin-based components allow elastic contraction and expansion, promote rapid endothelialization, and prevent both over-proliferation of smooth muscle cells and platelet activation (Waterhouse et al., 2010; Nguyen et al., 2016). The incorporation of synthetic elastin appears thus as a promising alternative to obtain remodelable vascular replacements with enhanced *in vivo* performance. In this regard, elastin-like recombinamers (ELRs) are powerful candidates to meet such demands. ELRs are tailored-made protein polymers based on the repetition of conserved motifs in tropoelastin, typically the pentapeptide Val-Pro-Gly-Xaa-Gly (VPGXG) (Urry et al., 1991). They combine the reproducibility of synthetic materials with the bioactivity and biocompatibility of biological ones, merging the best qualities of the “synthetic world” with the benefits of the “biological world”. Due to its bioinspiration in the sequence of the elastin, ELRs present outstanding elasticity and flexibility, being able to support stretch and recoil cycles with minimal fatigue, making them ideal candidates for engineering implants intended to experience repetitive movements (e.g. opening and closing cycles) (Urry et al., 1976). These protein-engineered polymers can prevent the formation of fibrotic tissue and promote the pro-healing type-2 macrophages over the pro-inflammatory type-1 macrophages (Ibáñez-Fonseca et al., 2020; González-Pérez F. et al., 2021). Moreover, their recombinant nature makes them a multivalent platform for the development of cell-instructive hydrogels for *in situ* TE with precise control of their properties and composition (Girotti et al., 2015). For example, multiple bioactive motifs (e.g., cell-adhesive motifs or protease-sensitive sequences among others) can be incorporated into their backbone, thus conferring enhanced cell migration and matrix remodeling capacities. Recent studies have explored the development of ELR hydrogels with protease cleavage sites and bioabsorbable properties, which demonstrated to favor stromal cell-colonization, degradation and remodeling upon the *in vivo* implantation (Flora et al., 2019; González-Pérez F. et al., 2021; Contessotto et al., 2021). This cell-mediated degradation capacity is highly interesting for an *in situ* approach, in which a balance between the scaffold degradation and the cell colonization and remodeling is required. These approximations further encompassed the use of the RGD (Arg-Gly-Asp) tripeptide, which conferred cell adhesive properties improving the biocompatibility and integration of the ELR-based scaffold. ELRs can be chemically decorated with catalyst-free click cross-linkable groups to guide the formation of stable hydrogels under mild physiological conditions through the strain-promoted [3 + 2] azide-alkyne cycloaddition (SPAAC) (Kolb et al., 2001). This technology has been exploited for the fabrication of ELR hydrogels with excellent properties for cardiovascular applications (e.g., vascular grafts (Fernández-Colino et al., 2019b; González-Pérez M. et al., 2021), coronary covered stents (de Torre et al., 2015; Fernández-Colino et al., 2019a), and more recently heart valves (Saidy et al., 2022), thus proving themselves as a very promising material for *in situ* TE.

Herein, we address for the first time the application of these advanced protein-engineered polymers to the fabrication of an elastin-like venous valve (EVV), suitable for *in situ* TE. Specifically, we propose a biohybrid concept (Neves et al., 2020), which combines the elastic and bioactive properties of the ELRs with the mechanical support of textile components. The textile component was embedded into a click-crosslinked hydrogel made of cell-adhesive and protease-sensitive ELRs, thus creating a cell-instructive scaffold that promotes endothelialization and prevents platelet activation. The biohybrid scaffold was then sutured into a braided stent manufactured from bioabsorbable magnesium, to enable minimal invasive implantation and avoid long-term adverse effects caused by a durable stent structure as a foreign body. Afterward, we performed *in vitro* characterization of the mechanical properties, hydrodynamic performance and cell response of the EVV. This study aims to set the basis for the fabrication of venous valve replacements from ELRs and provides insights into the development of other miniaturized elastin-based valves.

## Materials and methods

### ELR synthesis and modification

The ELRs used in this work, namely GTAR-ELR, RGD-ELR and VKV-ELR, have been previously described and their sequences can be found in Supplementary Table S1 (González-Pérez F. et al., 2021). The recombinamer GTAR-ELR was designed to contain the protease-sensitive sequence “GTAR”, which features a high affinity to the uPA enzyme. RGD-ELR includes RGD cell adhesion motifs, whilst VKV-ELR is a structural polymer (i.e. no bioactive nor protease-sensitive sequences are engineered within its backbone). The bioproduction of ELRs was performed following standard methods (Urry et al., 1991). Briefly, encoding genes were developed by using the cloning plasmid pDrive in *Escherichia coli* XL1 blue competent cells (Agilent, United States). The resulting constructs were cloned in pET25-derived plasmid and expressed in *E. coli* BLR bacteria (Agilent, United States). The bacterial fermentation was carried out in a 15-L bioreactor (Applikon Biotechnology, United States). The protein polymer purification was then performed through cooling and heating cycles (Inverse Transition Cycling), followed by centrifugation (McPherson et al., 1996). Finally, the aqueous ELRs solutions were dialyzed against ultra-pure water (Milli-Q, Sigma Aldrich, United States), filtered through 0.22 µm membranes for sterilization, and lyophilized. As regards chemical modification, azide groups were tethered to the RGD-ELR backbone, whilst cyclooctyne groups were attached to the GTAR-ELR and VKV-ELR backbones, thus obtaining the ‘click’ cross-linkable recombinamers required for hydrogel formation, as described elsewhere (González-Pérez F. et al., 2021).

### Fabrication of the elastin-like venous valve (EVV)

The recombinamers ELR-cyclooctyne (GTAR-ELR) and ELR-azide (RGD-ELR) were dissolved at 100 mg/ml in PBS/ethanol 1:1 (v/v) solution (Gibco, United States and Sigma Aldrich, United States, respectively) at room temperature (r.t.). After 1 hour, the corresponding ELRs were mixed in a 1:1 volume ratio and injected into the conical mold, which contains a polyethylene terephthalate (PET) textile mesh, previously heat-treated for 5 min at 170°C. The warp-knitted mesh was produced at *Institut für Textiltechnik* of the RWTH Aachen University (Germany), using a Karl Mayer, Double Raschel Warp Knitting Machine (DR 16 EEC/EAC, Germany). The PET multifilament displays 44 dtex/24f, with 36 yarns in the mesh and 21 effective arches per centimeter. The hexagonal shape of the textile cell was selected to provide the hybrid venous valve leaflets a higher degree of stretching in the radial direction than in the circumferential direction, which mimics the structural native valvular anisotropy (Supplementary Figure S1). PET was selected as a fabrication material of the textile due to its clinical record for the fabrication of large diameter vascular grafts (Zippel et al., 2001). While this material has also been associated with some issues, including long-term inflammation (Schlosser et al., 2002), the PET textile used for the EVVs is fully embedded in a ELR-matrix. Therefore, any potential inflammatory and thrombogenic issues triggered by PET are expected to be minimized thanks to the low platelet adhesion, endothelialization, and cell-mediated remodeling of the ELR matrix, during which the ELR will be degraded and replaced by newly synthesized ECM. The custom-made mold had a length of 45 mm, an upper diameter of 1.0 cm, a lower diameter of 1.2 cm and a wall thickness of 0.83 mm. After incubation for 1 h, the resulting biohybrid scaffolds (i.e. ELR hydrogels embedding the textile mesh) were demolded (Supplementary Figure S2A). Additionally, the possibility to fabricate an EVV with alternative ELRs (i.e. VKV-ELR + RGD-ELR) was assessed in parallel. Subsequently, the biohybrid scaffolds were washed with PBS overnight and sutured to the bioabsorbable magnesium stent structure. The stent (lumen diameter of 1.2 cm, height of 2.5 cm) was produced at the *Institut für Textiltechnik*, by braiding a WE43MEO magnesium-alloy-wire (Ø 300 µm) provided by Meotec GmbH (Germany). The stent featured an atraumatic design, with two symmetric stent segments and 12 deflection points at each side, and was further surface-modified by facilitating the Kermasorb^®^ plasma electrolytic oxidation (PEO) process (Meotec GmbH, Germany). In order to fashion the bileaflet EVV and integrate it into the stent, the biohybrid scaffolds were sutured at two opposite located points on the proximal edge, and along the circumferential line in the distal part.

### Equibiaxial mechanical properties of the EVV

The mechanical evaluation of the EVV was conducted on a biaxial tester (Zwick Roell Biaxial Tester, Spain) equipped with the analysis software (TestXpert II V3.71). Four arms with three hooks each were used to hold the sample in the radial and circumferential direction while testing (Supplementary Figure S3C). Four dots were marked inside the central region of each sample to capture the displacements with a coupled camera. The samples were kept moist in PBS and a 30 mN preload was applied in both directions. Subsequently, the samples were stretched equibiaxially up to 7.5 N at a strain rate of 1%/s for 20 loading-unloading cycles (to assure repeatability), taking the first five cycles as preconditioning tests. The 7.5 N force value was selected based on preliminary testing, in which 14 N and 24% strain resulted in the mechanical break for the EVV. A loading cycle to failure was performed at the end of the experiment, testing a total of n = 4 specimens per experimental group. The thickness of biohybrid constructs was measured by microscope images (VHX-5000 digital microscope, United States) using the ImageJ software. The collected data was used to obtain stress-strain curves, and the peak tangent modulus was calculated in the relatively linear region between 1 and 1.5 MPa stress.

### Burst strength analysis

The structural failure pressure of EVV was evaluated in a custom-made burst chamber consisting of a peristaltic pump (IPC Ismatec, Germany) and a pressure sensor (JUMO, Germany). Samples of 1 cm^2^ were placed in the chamber, and PBS was infused at a flow rate of 7.5 ml/min until structural failure. The data was recorded with a custom-made LabVIEW software (National Instruments, United States).

### Hydrodynamic testing of the EVV

The hydrodynamic testing of the EVV was conducted under controlled conditions in a custom-made bioreactor system. The bioreactor consists of a reservoir, a pulsatile pump 702-6882 (RS components, UK), an imaging chamber, and a silicone rubber flow loop (Ismatec, Germany) designed to mimic normal venous hemodynamic conditions (Lee et al., 1991). A 36.7% glycerol aqueous solution (Sigma Aldrich, United States) was used to replicate blood density (Midha et al., 2016). Peak flow rates ranging from 1 to 2 L/min and peak pressures ranging from 40 to 75 mmHg were tested to simulate the different conditions in the final implantation site. Pressure sensors Xtrans (Codan pvb Medical GmbH, Germany) were located before (distal) and after (proximal) the EVV. The set-up also included a flow sensor SonoTT™ Flowcomputer (Emtec GmbH, Germany). A custom-made LabVIEW software (National Instruments, United States) was used to record pressure, flow, and time. These values were further processed to calculate the percentage of regurgitation, medium open valve pressure drop during the open phase of the valve cycle, and closing times. Additionally, images were captured during valve testing with a camera (Olympus Stylus SH-1, Japan), and the effective orifice area (EOA) was calculated according to Eq 1 in cm^2^:

$$EOA=\frac{Q_{RMS}}{51.6\sqrt{\frac{\Delta P}{\rho }}}$$ (1)

Where Q_RMS_ is the root mean squared forward flow in mL/s during the positive differential pressure period (ΔP > 0), ΔP (mmHg) is the mean pressure difference during the positive differential pressure period (ΔP > 0) and ρ (g/cm^3^) is the density of the test fluid (1.058 g/cm^3^) (Stasiak et al., 2020). The percentage of EOA was calculated by diving by 0.785 cm^2^ (theoretical maximum orifice area of the valve) and multiplying by 100.

### Washout testing

The EVV was mounted in the hydrodynamic bioreactor with a peak pulsatile flow of 1,500 ml/min under peak column pressure of 60 mmHg. The 1,500 ml/min peak pulsatile flow that corresponds to 22.1 cm/s was selected to mimic the healthy physiologic conditions, reported between 13 and 41 cm/s in the 30-degrees head-up position (Moneta et al., 1988). For the test, dye pen solution in water 1:10 (v/v) was manually injected by two syringes placed on each of the two bellies of the leaflets, and images were acquired to analyze the dye washout from the injection site.

### *In vitro* simulated implantation procedure

The EVV was crimped and inserted in the delivery transcatheter device upon introduction of the expandable balloon (PTA dilatation catheter Atlas Gold, United States) through the center of the leaflets. Afterward, the valve was delivered into a silicon tube (Sigma Aldrich, United States) and the balloon was inflated up to a pressure of 6 atm with distilled water, expanding the valve up to 14 mm in diameter as advised by the manufacturer. Subsequently, the EVVs (n = 3) were tested in the bioreactor under a peak pulsatile flow of 1,500 ml/min and a peak column pressure of 60 mmHg.

Additionally, to evaluate the resistance of the valve after long runs, the EVV was subjected to repetitive mechanical stress-relaxation tests. First, the elastin-like hydrogel embedding the PET mesh was bent 100 cycles using tweezers and rolled around a metal cylinder, before being sutured to a self-expandable Nitinol stent (Wallstent-UniTM Endoprosthesis Boston Scientific, United States). Subsequently, the EVV was introduced in a crimping machine (Edwards THV Crimper 9600CR, United States), and subjected to 50 crimping cycles. Finally, the EVV was analyzed by scanning electron microscopy (SEM XL 30 FEG, Electron Microscopic Facility, Institute of Pathology, RWTH Aachen University Hospital, Germany) to evaluate the appearance of possible cracks and exposure of the PET mesh.

### Cell isolation and culture

Human umbilical cords were kindly provided by the RWTH Aachen University Centralized Biomaterial Bank (cBMB) according to its regulations, following RWTH Aachen University, Medical Faculty Ethics Committee approval (cBMB project number 323), after the written consent of three different donors at University Hospital Aachen (Germany). Subsequently, human umbilical vein endothelial primary cells (HUVECs) were isolated from veins of umbilical cords as reported previously (Moreira et al., 2015). HUVECs were cultured on 2% gelatin (Sigma-Aldrich, United States) coated flasks with endothelial cell growth medium (EGM) (PromoCell, Germany) supplemented with basic Fibroblast Growth Factor, Insulin-like Growth Factor, Vascular Endothelial Growth Factor 165, Ascorbic Acid, Heparin, Hydrocortisone and FCS (PromoCell, Germany). They were incubated at 37°C in a 5% CO_2_ humidified atmosphere. The medium was changed every 2 days and cells were used in passage three for subsequent experiments.

### *In vitro* endothelialization analysis

ELR hydrogels containing the textile mesh were sterilized in ethanol 70% (v/v), cut with a biopsy punch (6 mm diameter, 48,601 PFM Medical, Germany), washed in sterile PBS three times for 30 min at r. t. and incubated overnight in PBS in 96 well plates (VWR^®^, United States). The following day, 100 µL of HUVECs (6*10^5^ cells/mL, cell density 187,500 cells/cm^2^) were added on top of the hydrogels and cultured in a 5% CO_2_ humidified incubator for 24 h at 37°C.

After *in vitro* culture with HUVECs, samples were fixed in 4% formaldehyde (Carl Roth, Germany) in PBS at r. t. for 1 h, rinsed three times in PBS and permeabilized with 0.1% Triton™ X-100 (Sigma Aldrich, United States) for 5 min. Afterward, samples were incubated for 1 h at r. t. with 0.5% normal goat serum (Dako, Denmark) in 0.1% Triton™ X-100, followed by 45 min at r. t. and overnight incubation at 4°C with primary antibody mouse monoclonal anti-CD31 (endothelial cell labeling, 1:100 dilution, Sigma Aldrich, United States). The samples were washed with PBS (3 times) and incubated for 1 h at r. t. with goat anti-mouse IgG H&L secondary antibody conjugated to Alexa Fluor 594 (1:400, Invitrogen A11005, United States). Both antibodies were diluted in 1% Bovine Serum Albumin (Sigma Aldrich, United States) solution in PBS, supplemented with 0.1% sodium azide (Sigma Aldrich, United States). After rinsing three times with PBS, the samples were incubated in a solution of DAPI (1 μg/ml, Carl Roth, Germany) for 15 min, to stain cell nuclei in blue. Images from randomly chosen areas were obtained using EC Plan-Neofluar 10x and Plan Apochromat ×20 objectives in an inverted Zeiss LSM 710 confocal microscope (facility IZKF, RWTH Aachen University Hospital, Germany). Images were acquired by line sequential mode unidirectionally and saved as czi files. For DAPI visualization, the samples were excited with a 405 nm laser, and emission was collected at 410–521 nm, whereas for CD31 visualization, the samples were excited with a 561 nm laser, and emission was collected at 585–733 nm. The Zen black 2012 software (Carl Zeiss Microscopy GmbH, Germany) was used for image acquisition.

### Fibrin hydrogel formation

To create the fibrin hydrogels two different solutions were prepared. The solution A, which contains 30% (v/v) thrombin from bovine plasma (T4648 40 U/mL, Sigma Aldrich, United States), 40% (v/v) Tris-buffered saline (TBS) (pH 7.4, Sigma Aldrich, United States) and 30% (v/v) 50 mM calcium chloride dihydrate (Sigma Aldrich, United States) in TBS; and the solution B, which contains a 10 mg/ml solution of fibrinogen from human plasma (Calbiochem 341,576, Sigma Aldrich, United States) in TBS. The mixture of both solutions in a 1:1 volume ratio using a mixing nozzle (Automix-Tips, DMG, Germany) gives the corresponding fibrin hydrogels, after a minimum incubation time of 30 min at r. t.

### Thrombogenicity

Biohybrid scaffolds (i.e. ELR hydrogels embedding the textile mesh), as well as the fibrin and ePTFE (GORE-TEX^®^) controls, were washed 3 times in sterile PBS for 30 min at r. t. and stored in PBS overnight. The next day, human blood (drawn from healthy volunteers) with sodium citrate tribasic dihydrate 0.3% (Sigma Aldrich, United States) was centrifuged for 15 min at 2000 and 270 *g* in two different 50 ml tubes. Subsequently, a 1:1 mixture of the supernatants was added on top of the samples contained in the 96 well plates. After incubation at 37°C for 1 h, samples were fixated for 1 h in 3% glutaraldehyde solution (Carl Roth, Germany). Subsequently, samples were dehydrated by critical point drying, gold-sputtered with 12.5 nm Pd-Au alloy (EM SCD500, Leica, Germany) and observed by scanning electron microscopy (SEM XL 30 FEG, Electron Microscopic Facility, Institute of Pathology, RWTH Aachen University Hospital, Germany). High and low activated platelets were colorized using the Mountains software, courtesy of Digital Surf, France.

### Hemolytic analysis

ELR hydrogels embedding the textile mesh, as well as the fibrin and GORE-TEX^®^ controls, were washed 3 times in sterile PBS for 30 min at r. t. and stored in PBS overnight. The next day, a 9:1 solution of PBS and human blood containing sodium citrate at 0.03% was centrifuged and the supernatant was discarded. This step was repeated until the supernatant was clear. Subsequently, a 3% w/v solution of red blood cells in PBS was prepared and added (200 µL) on top of the samples contained in the 96 well plates, followed by incubation at 37°C for 4 h in a humidified chamber. A 3% w/v solution of red blood cells in deionized (DI) water was used as a positive control. Finally, samples were centrifuged at 10,000 rpm for 10 min, the supernatant was collected and the absorbance was measured at 540 nm in a spectrophotometer (Tecan infinite 200, Switzerland). Eq. (2) was applied for the evaluation of the % of hemolysis:

$$Hemolysis\;\left(\%\right)=\frac{\left(\text{AbsSample}-\text{AbsPBS}\right)}{\left(\text{AbsDIWater}-\text{AbsPBS}\right)}x\;100$$ (2)

### Statistical analysis

Statistical differences were analyzed using a one-way analysis of variance (ANOVA) followed by the post-hoc Holm–Sidak method. All the experiments were performed at least in triplicate (n ≥ 3). Values were expressed as mean ± standard deviation, accepting as statistically significant a *p*-value < 0.05. (**) indicates *p* < 0.001 and (*) *p* < 0.05, while n. s indicates no significant differences (*p* > 0.05).

## Results and discussion

### Elastin-like venous valve (EVV) fabrication and characterization

**FIGURE 1:**
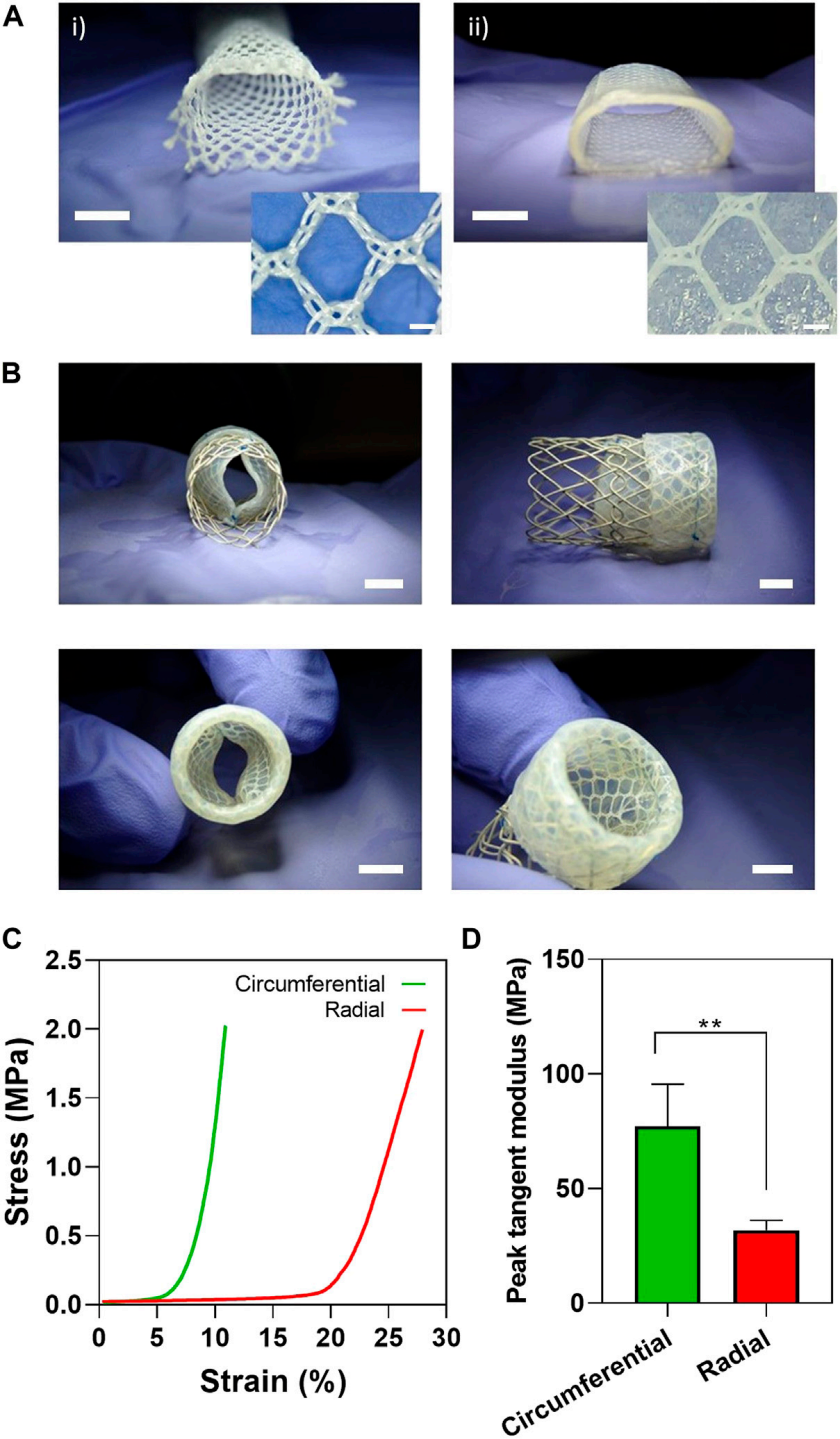
Biohybrid EVV fabrication and mechanical characterization by equibiaxial tensile testing. **(A)** (I) Wrap-knitted tubular textile and (ii) biohybrid scaffold (wrap-knitted tubular textile embedded with ELR hydrogel). Scale bar = 5 mm for (i) and (ii), and 500 µm for the small inserts. **(B)** Bileaflet EVV obtained by suturing the biohybrid tubular construct into the magnesium stent. Scale bar = 5 mm for left pictures and 4 mm for right pictures. **(C)** Representative stress-strain curves for the EVV tested in the circumferential and radial directions. **(D)** Peak tangent moduli measured between one and 1.5 MPa of stress, represented as mean ± SD (**) p < 0.001.

We successfully embedded a wrap-knitted tubular textile with an elastin-like hydrogel matrix by using a simple injection molding approach combined with the catalyst-free click-chemistry (Figure 1A). Specifically, the selected ELR scaffold (Supplementary Table S1) contained specific bioactive motifs (i.e. the uPA-specific cleavage GTAR motif and the cell adhesion RGD tripeptide) and has previously been demonstrated to favor cell-colonization and implant remodeling into native-like tissue *in vivo* (Flora et al., 2019; González-Pérez F. et al., 2021). The textile-reinforced elastin-based tubular construct was integrated into the magnesium stent and fashioned into a bileaflet venous valve by suturing at specific points (Figure 1B). This biohybrid scaffold was able to bend 180°, without any holes or voids. This was achieved thanks to the unique combination of the flexibility of the ELRs with the stability provided by the reinforcement.

A venous valve prosthesis has to withstand the vascular pressures and ideally reproduce the mechanical behavior of the native venous valves. In order to evaluate the mechanical response of the EVV, equibiaxial tensile testing and burst strength tests of the textile-reinforced elastin-like hydrogels were carried out. Stress-strain curves revealed (i) radial to circumferential anisotropy, matching the textile design (Supplementary Information, Figure S1) and (ii) a marked nonlinear response, analogous to the J-shaped profile characteristic of native valve tissues (Huang and Lu, 2017; Lu and Huang, 2018; Benson and Huang, 2019; Saidy et al., 2022). Specifically, three distinct regions were identified within the exponential stress-strain curves (Figure 1C): (i) a fairly linear region at the beginning (with low tangent modulus), followed by (ii) a non-linear transition region, and (iii) relatively a linear region at the highest values of strain, with the highest tangent modulus.

The peak tangent modulus in the circumferential direction was 77 ± 18 MPa, which was approximately twofold higher than the value displayed in the radial direction, i.e. 32 ± 4 MPa (Figure 1D). These values were comparable with those displayed by the bovine saphenous venous valve (77 ± 25 MPa in the circumferential and 36 ± 20 MPa in the radial direction, the latter resulted from the averaged values of proximal and distal valvular tissue) (Lu and Huang, 2018). This mimicry in the mechanical performance between the native bovine saphenous valves and the EVV sets up the first step towards the successful performance of the valve upon implantation *in vivo*. Ackroyd et al. reported ultimate tensile strengths of ∼9 MPa and percentage of strain of ∼35% (breaking stress and strains) for human femoral vein valve leaflets, measured by uniaxial testing (Ackroyd et al., 1985). These values approximate our EVV measurements (5.55 ± 0.75 MPa of stress (radial direction), 6.44 ± 2.51 MPa of stress (circumferential direction), 30.9 ± 1.37% of strain (radial direction), and 17.4 ± 4.31% of strain (circumferential direction)) (Supplementary Table S2). However, a direct comparison with these values is challenging, as the applied stress by Ackroyd and colleagues was limited to one single direction (uniaxial testing), which is also less representative of the native scenario.

Additionally, we performed burst strength tests to confirm whether our venous valve substitute withstands the hemodynamic pressure conditions required for safe implantation in the circulatory system. The burst analysis of the EVV showed a structural failure at a pressure of 333 ± 38 mmHg (Supplementary Figure S4A). This value is 3 times higher than the physiological static venous pressure near the foot, which is normally between 90 and 100 mmHg (Sathe and Ku, 2007). Notably, this value meets the criteria of Sathe et al., who proposed that a venous valve should be able to hold 300 mmHg of backpressure without damage, and points to the suitability of the valve to withstand the peak stresses generated in changes from supine to upright position (Sathe and Ku, 2007).

In order to check the feasibility to apply this fabrication approach to other ELRs, we also fabricated and characterized tubular constructs with an alternative ELR combination (i.e. structural VKV-ELR and bioactive RGD-ELR, Supplementary Figures S2B, C). The resulting venous valve also showed nonlinear anisotropic behavior with values similar to those of the native bovine saphenous vein (57 ± 4 MPa and 29 ± 5 MPa for the circumferential and radial directions, respectively), and a burst strength (322 ± 28 mmHg) that comply with the required standards (Supplementary Figures S3A, B; Supplementary Figure S4B). This demonstrates the versatility of the applied strategy, which is not limited to a specific ELR and can be applied to ELRs with different functionalities, tailorable *ad-hoc* by recombinant methods.

### Hydrodynamic testing of the EVV in a pulse simulator system

**FIGURE 2:**
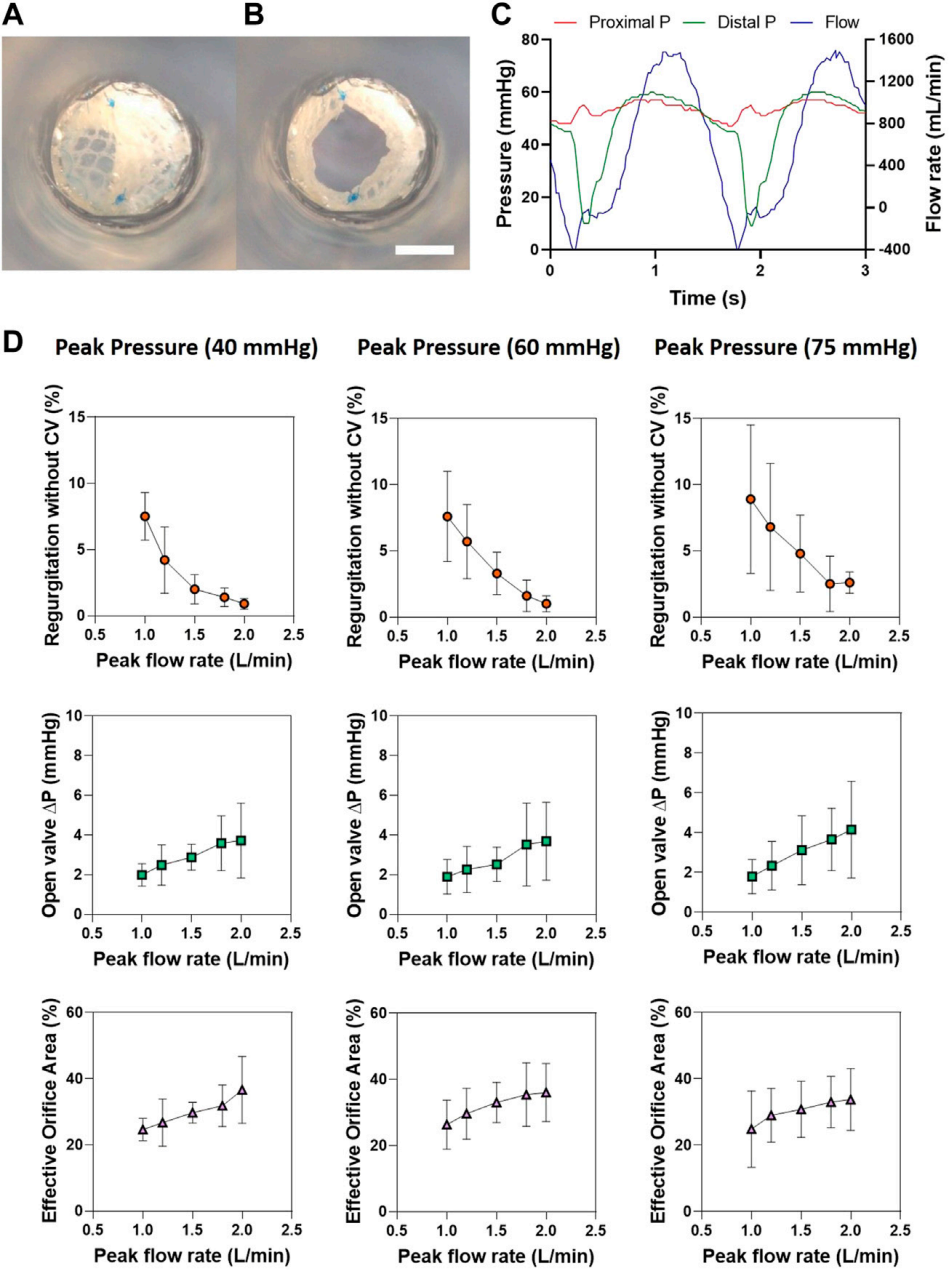
Hydrodynamic testing of the EVV in the pulse duplicator system. **(A,B)** Representative images of the EVV in its open and close state. Scale bar = 5 mm **(C)** Pressure-flow profiles for the EVV tested at peak column pressure of 60 mmHg and peak flow rates of 1,500 ml/min. **(D)** Regurgitation without closing volume, EOA (%), and open-valve pressure drop were tested at different pressure and flow rates.

To assess whether the EVV withstands the physiological hemodynamic conditions of the circulatory system, we used a pulsatile bioreactor. Representative frames of the valve in the open and close position and pressure-flow curves of the EVV tested at 60 mmHg peak pressure and 1,500 ml/min peak flow rate appear in Figures 2A–C. Results regarding regurgitation, pressure drop and EOA (%) at the three different peak pressures (40, 60 and 75 mmHg) and five different peak flow rates tested (1,000, 1,200, 1,500, 1,800 and 2,000 ml/min) are shown in Figure 2D.

The EVV showed excellent hydrodynamic performances for all the tested conditions. Specifically, the regurgitation values were below 9%, and decreased by increasing the peak flow rates, as expected. This performance matches the requirements proposed by Tanner et al. for functional venous valves, in which the regurgitant threshold is fixed at 10% (extrapolated from heart valve ISO-5840 standards) (Tanner, 2013). Open-valve pressure drops were lower than 5 mmHg, thus meeting the value (<5 mmHg) proposed by Sathe et al. (Sathe and Ku, 2007). The EOA (%) ranged between 20 and 40%, with a slight tendency to increase by increasing the peak flow rate. No limits are described for EOA (%) of venous valves. For heart valves, the minimum values required by ISO standards are 0.85 cm^2^ for 19 mm and 1.05 cm^2^ for 21 mm, which corresponds to an EOA (%) of 30% in both cases (Moreira et al., 2015; Stasiak et al., 2020). In addition, closing times showed values <0.4 s, being lower than the 0.5 s reported for a functional venous valve by Tanner et al., thus indicating its potential as a venous valve replacement (Tanner, 2013) (Supplementary Figure S5E).

**FIGURE 3:**
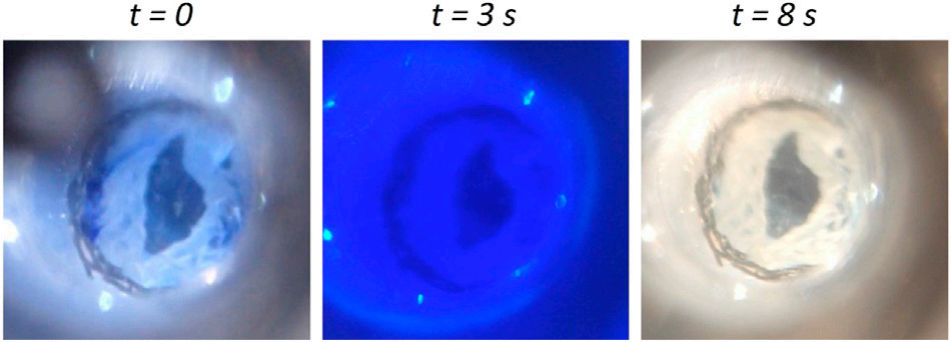
Washout testing of the EVV. Representative images of the EVV at the ink-injection time, after 3 s and after complete wash-out (8 s). The test was performed at a peak pulsatile flow rate of 1,500 ml/min and a peak pressure of 60 mmHg.

Additionally, the EVV showed a rapid washout, and no stagnation points were detected behind the leaflets upon ink injection, during a peak pulsatile flow rate of 1,500 ml/min (Figure 3). This lack of dye deposition indicates a proper clearance in the belly of the valve, which is a pivotal aspect to minimize blood clotting and thrombus formation (Ryo et al., 1976).

Notably, the alternative version of the EVV (Supplementary Material) showed values of regurgitation, open-valve pressure drops, EOA (%), closing times and wash-out times (8 s) within the standard values stated for functional venous valves, which evidences the universality of the recombinant fabrication approach (Supplementary Figures S5A–D).

### *In vitro* cell response of the EVV: hemocompatibility and endothelialization

The fabrication of a venous valve intended as an *in situ* TE implant requires the use of a hemocompatible material capable to avoid thrombogenesis upon implantation. Therefore, we evaluated the reaction of the EVV to blood components by thrombogenicity and hemolysis assays.

**FIGURE 4:**
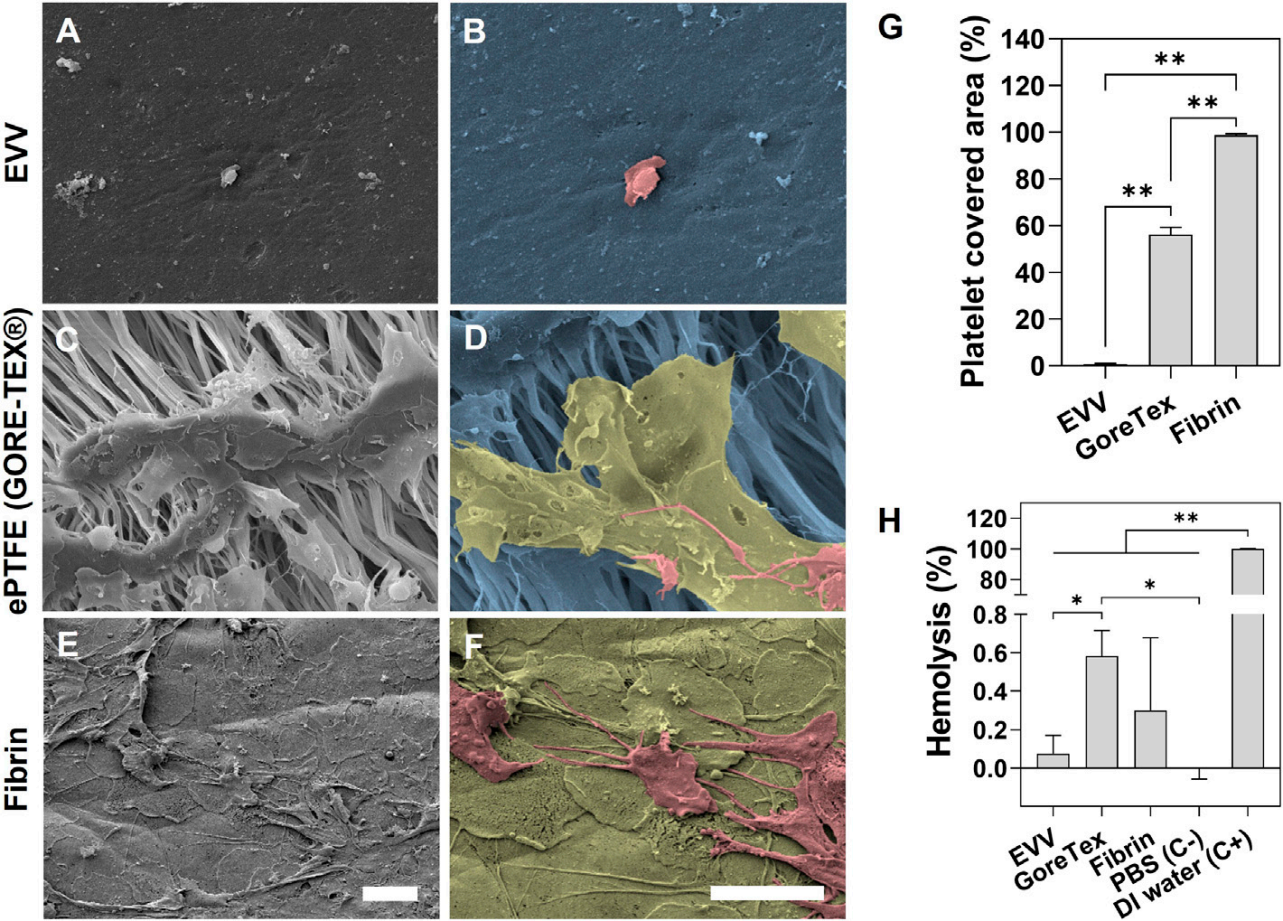
Hemocompatibility assessment. Representative SEM images of **(A,B)** EVV, **(C,D)** GORE-TEX^®^ and **(E,F)** fibrin after platelet adhesion study. **(B)**, **(D)** and **(F)** show zoom-in SEM images of EVV, GORE-TEX^®^ and fibrin respectively. Herein, the fully activated platelets with “fried egg” morphology were colored in green, the mild activated and non-activated platelets with extended pseudopodia or round morphology were colored in salmon, and the material surfaces were colored in blue. Scale bar = 5 µm. **(G)** Percentage of platelet covered area, represented as mean ± SD. **(H)** Percentage of hemolysis and comparison with GORE-TEX^®^, fibrin, and negative and positive controls, represented as mean ± SD. (*) p < 0.05 and (**) p < 0.001.

SEM images of EVV after incubation with platelet-rich plasma revealed a minimal amount of platelet adhesion in contrast to fibrin and GORE-TEX^®^ used here as controls (Duque and Larraga, 2012) (Figures 4A–F). In addition, the platelets on the surface of the EVV showed a round morphology, which indicates a low or non-activated state. On the other hand, the platelets present in fibrin and GORE-TEX^®^ controls appeared with a “fried egg” morphology (fully activated, colored in green) or a spread morphology with extended pseudopodia (mild activated, colored in salmon). Further quantification analysis showed that EVV samples had a platelet covered area ∼60 and 99% smaller than GORE-TEX^®^ and fibrin controls, respectively (Figure 4G). Additionally, the EVV, as well as fibrin and GORE-TEX^®^, showed negligible percentages of hemolysis, with 0.06 ± 0.09%, 0.30 ± 0.38%, and 0.58 ± 0.13% respectively, with the EVV samples showing even lower values than GORE-TEX^®^ (Figure 4H).

**FIGURE 5:**
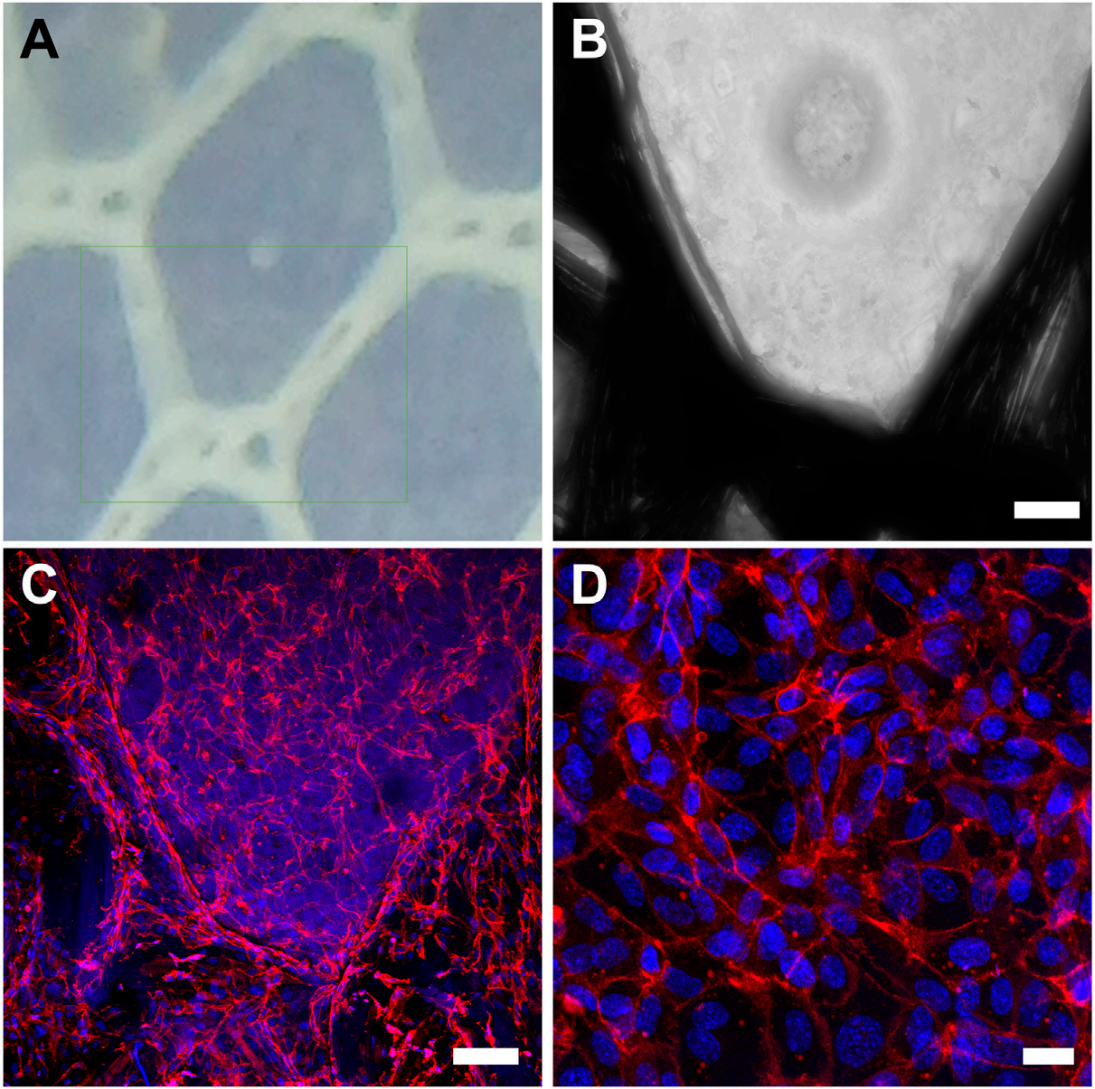
*In vitro* endothelialization study. **(A)** and **(B)** close-up images of the textile embedded with ELRs. **(C)** and **(D)** Representative confocal images of the EVV after 1 day of culture with HUVECs and subsequent immunostaining for CD31 and counterstaining with DAPI. Scale bar = 100 µm for **(B,C)**, and scale bar = 20 µm for **(D)**.

We then examined the ability of the EVV to promote endothelialization, given its importance upon implantation (Jana, 2019). We incubated the EVV with HUVECs for 24 h, and subsequently immunostained for CD31 and counterstained with DAPI. Confocal microscopy images confirmed that HUVECs attached and spread on the construct and formed a confluent layer (Figure 5). Images were taken from randomly chosen areas, which were representative of the entire sample. This behavior is correlated with the RGD cell adhesion motifs present in the ELR, which provide suitable anchor points for cell attachment and proliferation, as previously reported in *in vitro* experiments, in which HUVEC adhesion occurred only after 2 h (Fernández-Colino et al., 2019b, 2019a; González-Pérez F. et al., 2021).

The alternative version of the EVV also showed a minimal amount of platelet adhesion, with a covered area of only 1.0 ± 0.2%, which contrast with the behavior of GORE-TEX^®^ (56.1 ± 3.1% covered area) and fibrin (full covered area 98.8 ± 0.6%) and hemolysis value of 0.06 ± 0.11%. Since it also presented RGD motifs, the rapid endothelialization was also facilitated (Supplementary Figure S6), demonstrating the applicability to other ELR recombinant designs.

These results indicate the suitability of ELRs for the fabrication of cell-free cardiovascular implants, being in agreement with the data found in the literature (de Torre et al., 2015; Fernández-Colino et al., 2019b, 2019a). These advanced elastin-like biomaterials, used here for the first time to develop a transcatheter venous valve for *in situ* TE (Supplementary Figure S7), have the potential to alleviate the thrombogenesis complications present in other venous valve concepts, in which the fabrication material was associated with hemocompatibility issues (Gale et al., 1999; Pavcnik et al., 2008).

### *In vitro* simulated implantation procedure

**FIGURE 6:**
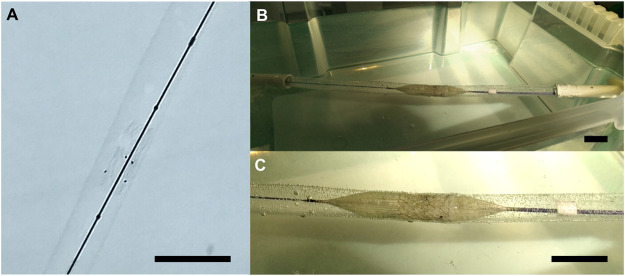
Representative images of the EVV in the balloon expansion transcatheter delivery. **(A)** EVV prior to balloon expansion, with four tantalum tubes sutured to facilitate X-ray visualization. **(B)** EVV upon balloon expansion. **(C)** Enlarged view of the EVV upon balloon expansion. Scale bar = 2.5 cm.

To test the suitability of the EVV for transcatheter implantation into the venous system, an *in vitro* simulated delivery procedure was carried out. The simulated delivery of the EVV into a 12 mm silicon tube demonstrated an expansion without macroscopic nor microscopic rupture of the biohybrid scaffold upon balloon dilatation from 6 to 14 mm diameter (Figure 6), in accordance with previous balloon-expansion studies performed with ELR constructs (de Torre et al., 2015). Subsequent testing of the EVV in the hydrodynamic bioreactor at 1,500 ml/min peak pulsatile flow and 60 mmHg peak pressure, showed a small detriment in performance in comparison with that before the delivery test, but still in accordance with values stated for functional venous valves (Supplementary Figure S8; Supplementary Table S3). This highlights the elastic performance and stability of the valve upon typical clinical handling procedures.

Additionally, the ELR embedding the PET textile did not show any crack areas nor the exposure of PET after repetitive bending cycles (Supplementary Figure S9). The suitability of the ELR embedding was further corroborated by subjecting the EVV to repetitive crimping (upon suturing to a Nitinol stent), followed by SEM visualization (Supplementary Figures S10, S11). These results make patent the outstanding elasticity of the EVV valve, and set an important step forward toward their future evaluation *in vivo*.

## Conclusion

ELRs engineered with different functionalities, together with a textile mesh and a bioabsorbable magnesium stent, allowed the fabrication of ready-to-use cell-free transcatheter venous valve substitutes for CVI. The resulting EVV featured native-like anisotropy and peak tangent moduli, besides being able to withstand pressures two to three times higher than the physiological values. *In vitro* studies supported the non-thrombogenic, hemocompatible and pro-endothelialization properties of the fabricated constructs. The hemodynamic analysis identified the excellent performance of the EVVs under pulsatile conditions, thus complying with values stated for functional venous valves. The washout studies confirmed the absence of stagnation points and the maintenance of valve functionality upon *in vitro* simulated transcatheter delivery procedure. Overall, this work provides an insight into the development of bioabsorbable ready-to-use venous valve substitutes that, after future animal *in vivo* studies, may offer a clinically relevant solution for CVI treatment.

## Data Availability

Data associated with this study is available upon reasonable request to the corresponding authors.
