# Supplementary material for: Biohybrid elastin-like venous valve with potential for in situ tissue engineering
Source: Front Bioeng Biotechnol. 2022 Sep 21;10:988533. doi: 10.3389/fbioe.2022.988533 (PMC9532864; doi:10.3389/fbioe.2022.988533)
Supplement: Supplementary file 1 [file DataSheet1.pdf]

Supplementary Material

Table S1. Amino acid sequence and molecular weight (MW) of the ELRs used for the fabrication of the venous valve. The sequence corresponding to the cell adhesion motif is represented in red, whereas the protease-sensitive sequence to the serine protease uPA is denoted in green.

	**Abbreviated amino acid sequence**	**MW (Da)**
**RGD-ELR**	MGSSHHHHHHSSGLVPRGSHMESLLP([(VPGIG)$_2$(VPGKG)(VPGIG)$_2$]$_2$ AVTG**RGD**SPASS[(VPGIG)$_2$(VPGKG)(VPGIG)$_2$]$_2$)$_6$V	60650
**GTAR-ELR**	MESLLP([(VPGIG)$_2$VPGKG(VPGIG)$_2$]$_2$YAVTG**GTAR**SASPASSA [(VPGIG)$_2$VPGKG(VPGIG)$_2$]$_2$)$_4$V	41052
**VKV-ELR**	MESLLPVGVPGVG[VPGKG(VPGVG)$_5$]$_{23}$VPGKG(VPGVG)$_3$VPGV	60451

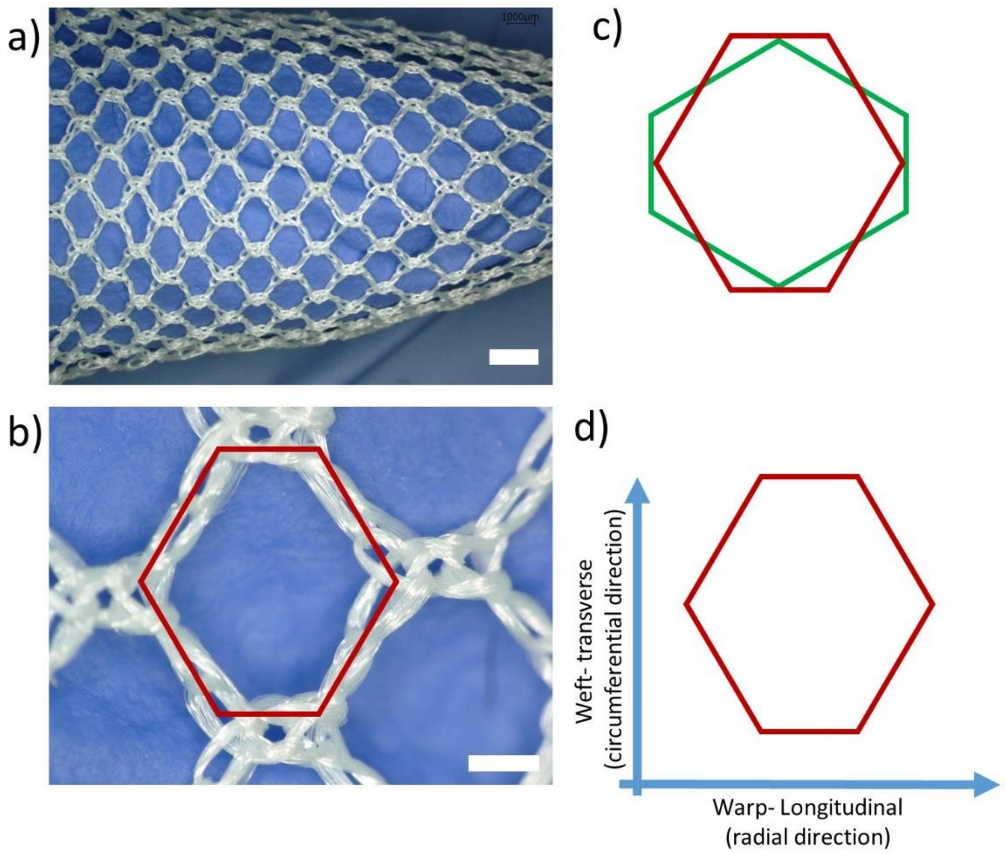

Figure S1. Anisotropic warp-knitted textile. (a) Overview. Scale bar = 2 mm. (b) Detailed view of the hexagonal shape of the textile “cell”. Scale bar = 500 µm. (c) Superposition of two hexagonal cells of the textile, one turned 90° with respect to the other. As shown in the scheme, there is no overlapping between both cells, which evidences the structural anisotropy of the textile. (d) Schematic indicating the warp and weft direction of the textile.

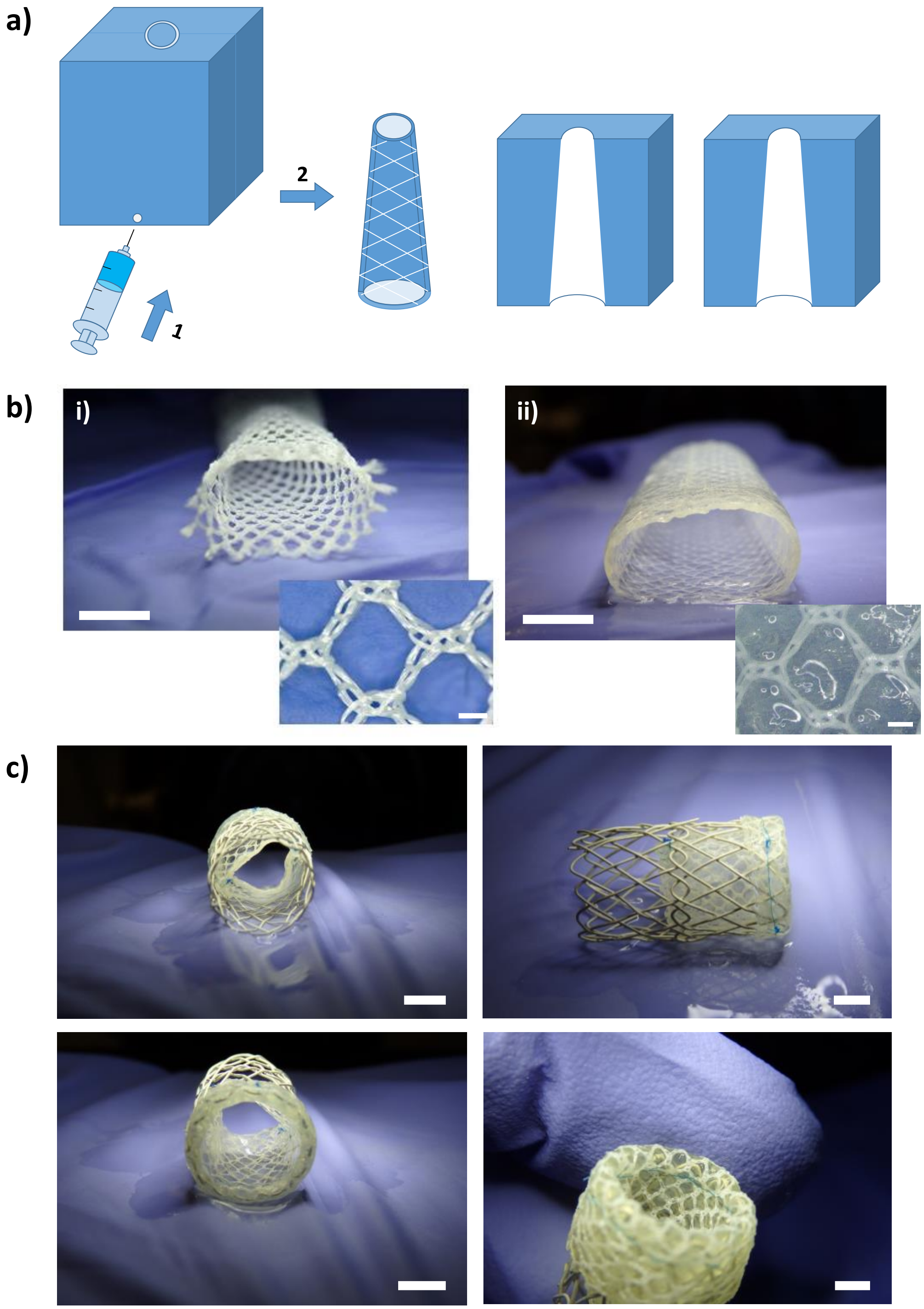

Figure S2. (a) Representative steps of the EVV fabrication. (1) First, the corresponding ELR mixtures (GTAR-ELR and RGD-ELR or VKV-ELR and RGD-ELR) are injected into the conical mold containing the textile mesh and incubated for 1 h. (2) Secondly, the two parts of the mold are detached obtaining the ELR scaffolds embedding the textile mesh. (b) Representative image of the (i) wrap-knitted tubular textile and (ii) the ELR hydrogel fabricated with VKV-ELR+ RGD-ELR, embedding

the textile mesh after the fabrication process. Scale bar = 5 mm for (i) and (ii), and 500 µm for the small inserts. (c) Representative images of the bileaflet EVV manufactured with VKV-ELR+ RGD-ELR, fashioned by suturing the biohybrid tubular construct to the magnesium degradable stent. Herein, two opposite commissural points are sutured to create the two leaflets following single point attached commissures (SPAC) technique, in addition to a circumferential line to fix the biohybrid scaffold to the distal position of the stent. Scale bar = 5 mm.

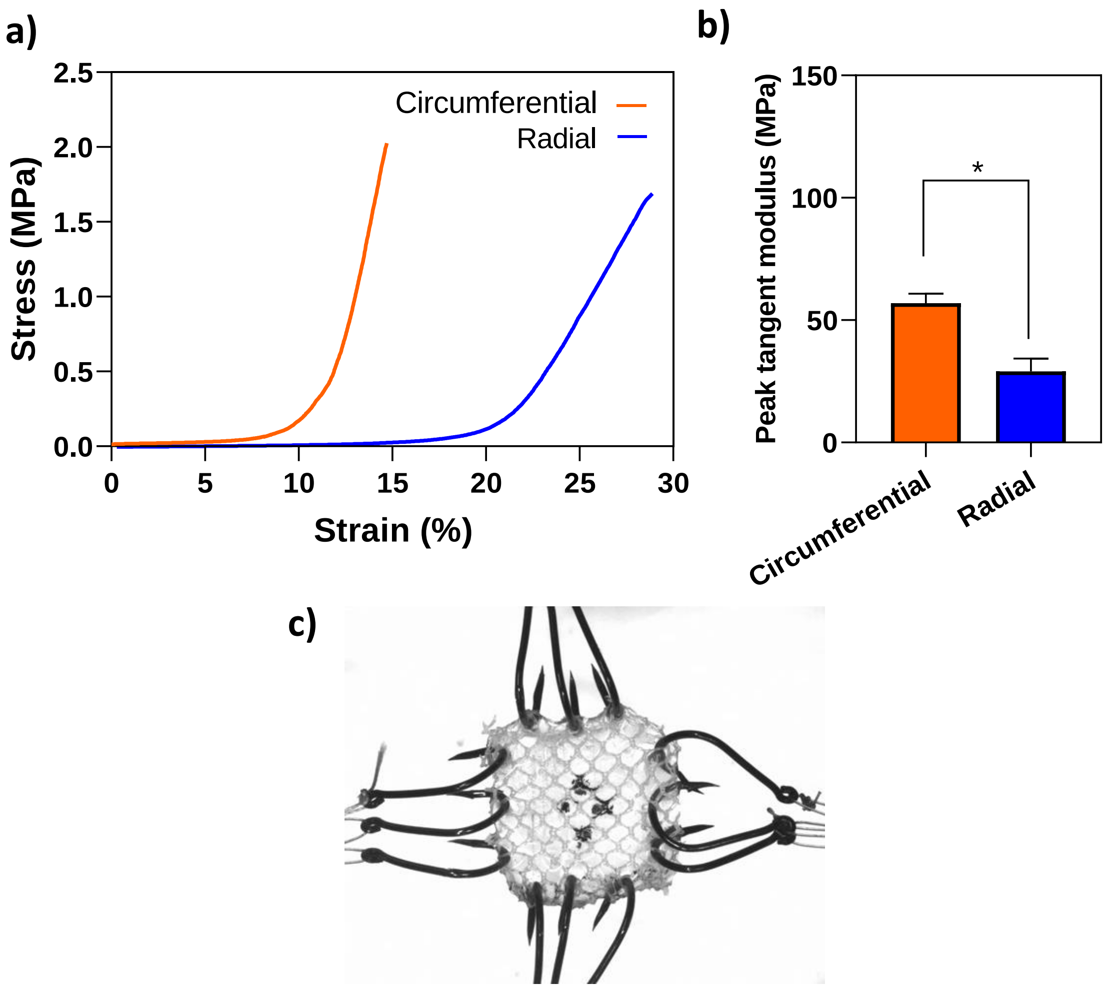

Figure S3. Mechanical characterization by equibiaxial tensile testing. (a) Representative stress-strain curves for the EVV manufactured with VKV-ELR+ RGD-ELR, tested in the circumferential and radial directions. (b) Peak tangent moduli represented as mean ± SD. (*) $p < 0.05$. (c) Representative image of equibiaxial testing.

Table S2. Mechanical properties of the EVV and native venous valves reported in literature.

**Tested tissue/ implant**	**Type of tensile testing**	**Mechanical Values reported**		**Ref.**
		**Peak tangent modulus**	**Ultimate tensile strengths (at breaking)**	
Biohybrid elastin-like venous valve (EVV)	Biaxial	- Circumferential: 77±18 MPa<br>- Radial: 32±4 MPa	- Circumferential: 6.44±2.51 MPa, and percentage of strain of 17.4±4.31 %<br>- Radial: 5.55±0.75 MPa, and percentage of strain of 30.9±1.37 %	Present study
Fresh human femoral venous valve leaflets	Uniaxial	Not reported	~9 MPa and percentage of strain of ~35%	(Ackroyd et al., 1985)
Bovine saphenous venous valve leaflets	Biaxial	▪ For the proximal valve:<br>- Circumferential (62.7±34.9 MPa)<br>- Radial (41.6±16.0 MPa)<br>▪ For the distal valve:<br>- Circumferential (92.1±27.9 MPa)<br>- Radial (31.8±13.9 MPa)	Not reported	(Lu and Huang, 2018)

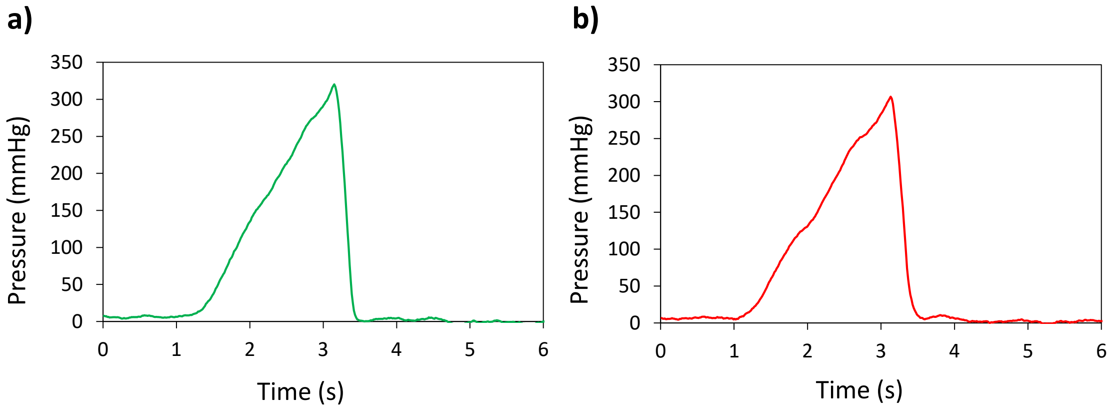

Figure S4. Representative burst strength curves of (a) EVV, manufactured with GTAR-ELR+ RGD-ELR and (b) EVV, manufactured with VKV-ELR+ RGD-ELR.

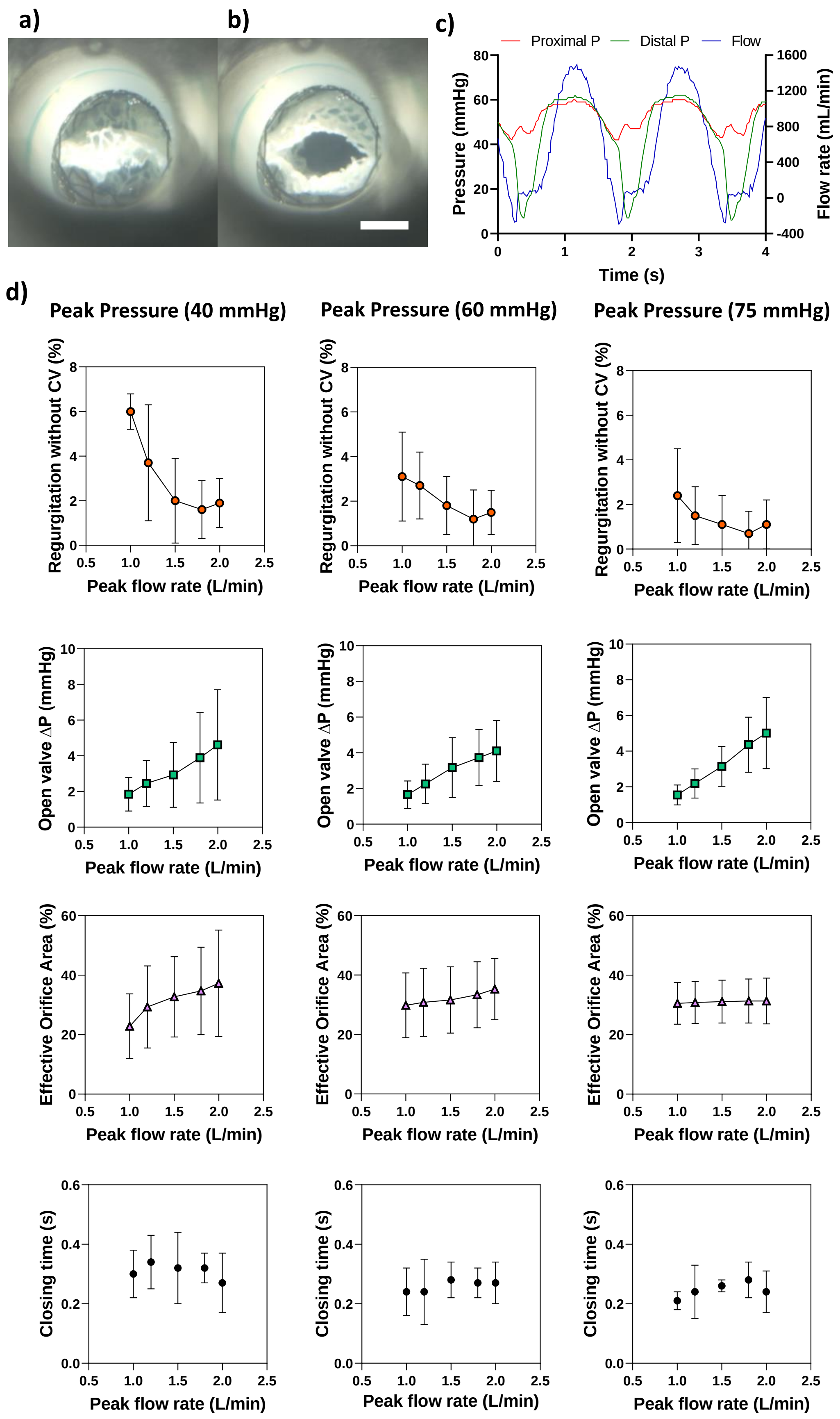
a)
b)
c)
Proximal P
Distal P
Flow
Pressure (mmHg)
Flow rate (mL/min)
Time (s)
d)
Peak Pressure (40 mmHg)
Peak Pressure (60 mmHg)
Peak Pressure (75 mmHg)
Regurgitation without CV (%)
Open valve ΔP (mmHg)
Effective Orifice Area (%)
Closing time (s)
Peak flow rate (L/min)

e)

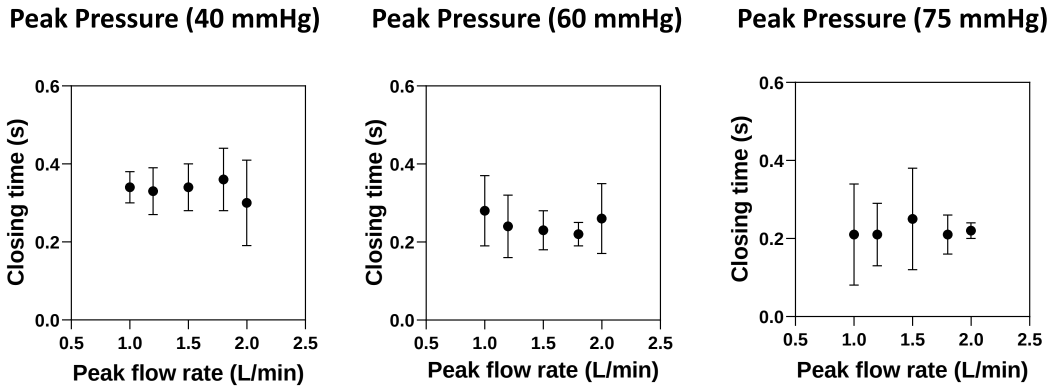

Figure S5. Hydrodynamic testing of the EVVs in the pulse duplicator system. (a,b) Representative images of the EVV manufactured with VKV-ELR+ RGD-ELR in their open and close state. Scale bar = 5 mm (c) Pressure-flow profiles for the EVV manufactured with VKV-ELR+ RGD-ELR, tested at peak pressure conditions of 60 mmHg and peak flow rates of 1500 mL/min. (d) Values of regurgitation without closing volume, effective orifice area (%), open-valve pressure drop and closing times at the different pressure and flow rate conditions of the EVV manufactured with VKV-ELR+ RGD-ELR. (e) Closing times for the EVV at the different pressure and flow rate conditions.

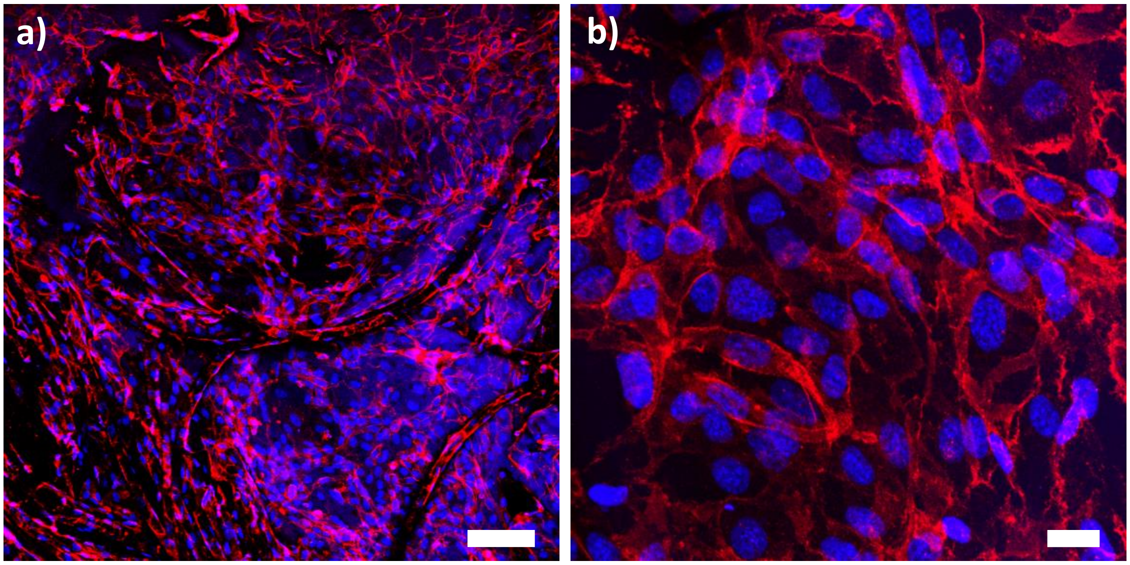

Figure S6. *In vitro* endothelialization study. Confocal images of the EVV manufactured with VKV-ELR+ RGD-ELR after 1 day of culture with HUVECs, and subsequent immunostaining for CD31 and counterstaining with DAPI. Scale bar = 100 µm for (a), and scale bar = 20 µm for (b).

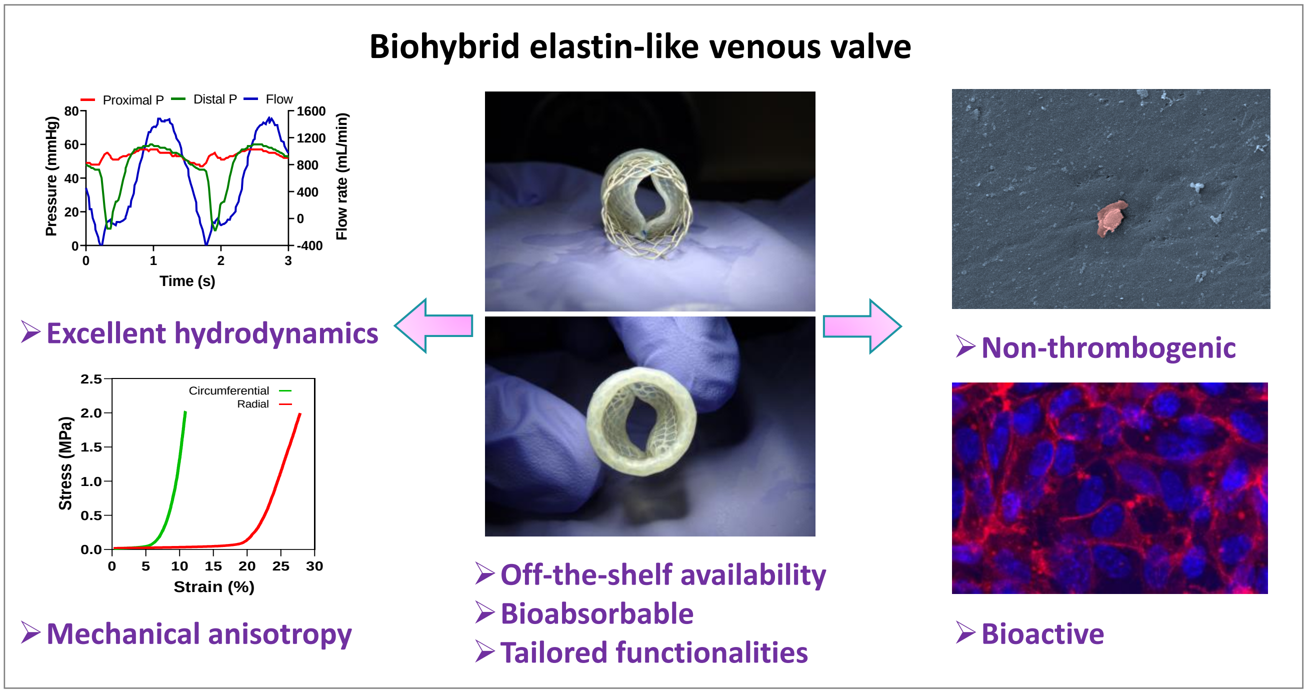

Figure S7. Schematic overview of EVV properties that support its potential for CVI treatment.

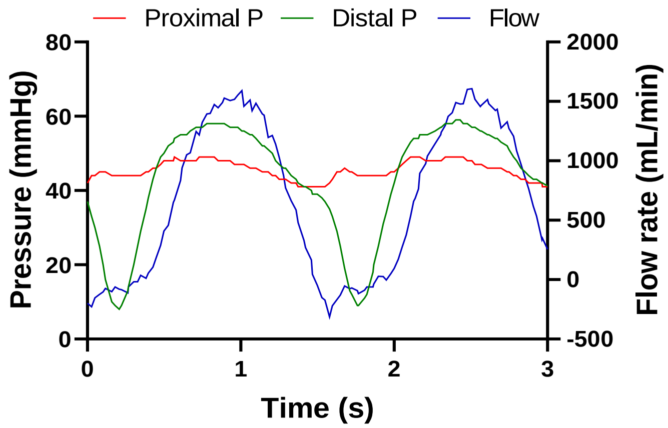

Figure S8. Representative pressure-flow profile of the EVV (n = 3) at peak pressure conditions of 60 mmHg and peak flow rate of 1500 mL/min, tested after *in vitro* simulation of transcatheter delivery.

Table S3. Corresponding regurgitation without closing volume, open-valve pressure drop, effective orifice area and closing times for the EVV, tested at peak pressure conditions of 60 mmHg and peak flow rates of 1500 mL/min, after *in vitro* simulation of transcatheter delivery, showing a small detriment in performance but still in accordance with values stated for functional venous valves.

	**Regurgitation without CV (%)**	**Open valve ΔP (mmHg)**	**Effective Orifice Area (EOA) (%)**	**Closing time (s)**
Average ± SD	4.6 ± 3.5	4.0 ± 1.6	26.3 ± 4.5	0.35 ± 0.07

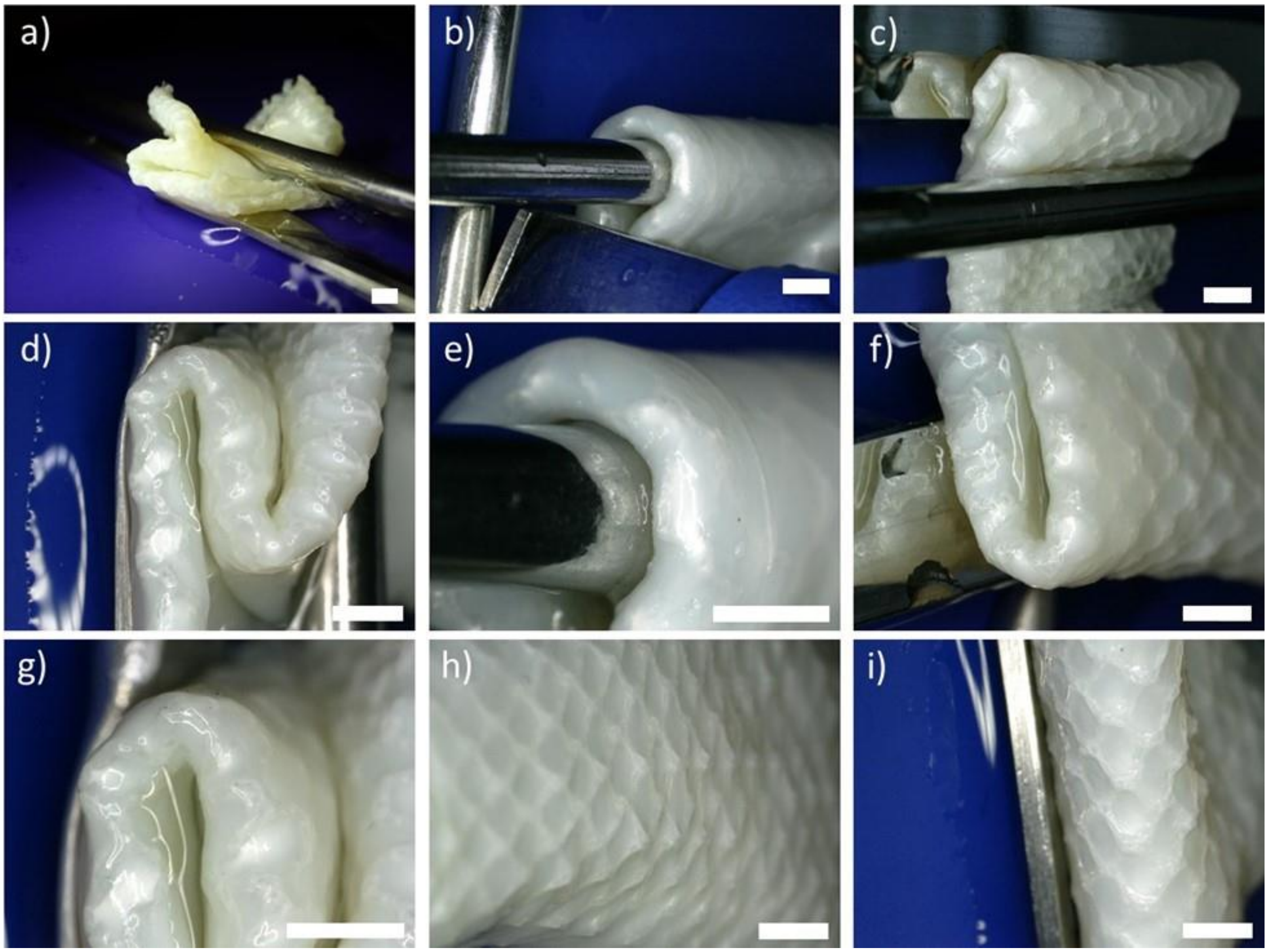

Figure S9. Repetitive mechanical stress-relaxation testing of the elastin (GTAR-ELR+ RGD-ELR) embedding the PET mesh. (a-i) Representative images after 100 bending cycles using tweezers and a metal cylinder. Scale bar = 2 mm.

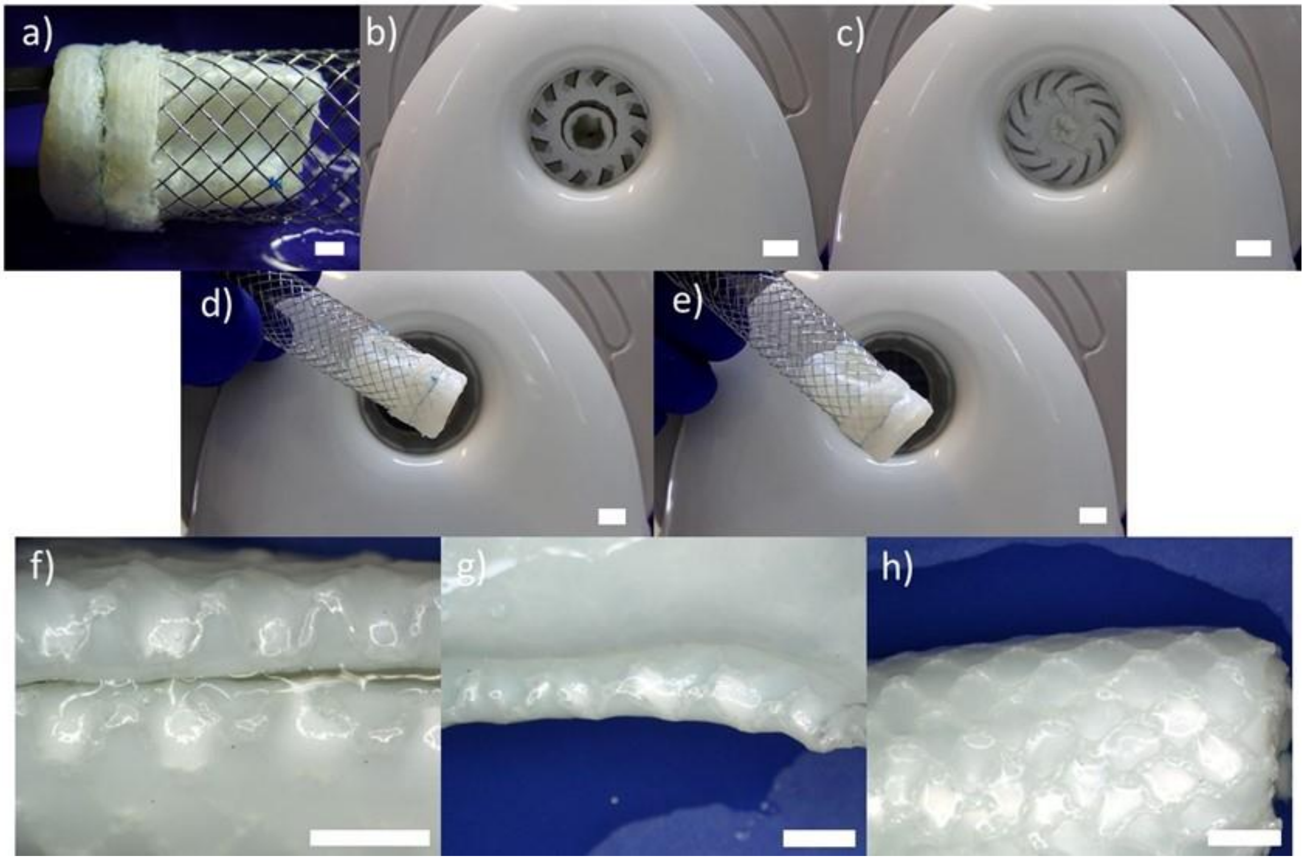

Figure S10. Repetitive mechanical stress-relaxation testing of the EVV. (a) Representative image of the elastin (GTAR-ELR+ RGD-ELR) embedding the PET mesh sutured to a Nitinol self-expandable stent after the 100 bending cycles testing. Scale bar = 2 mm. (b,c) Representative images of the EVV during the crimping tests. Scale bar = 1 cm. (d-h) Representative images of the EVV after 40 crimping cycles. (d,e) Scale bar = 5 mm. (f-h) Scale bar = 2 mm.

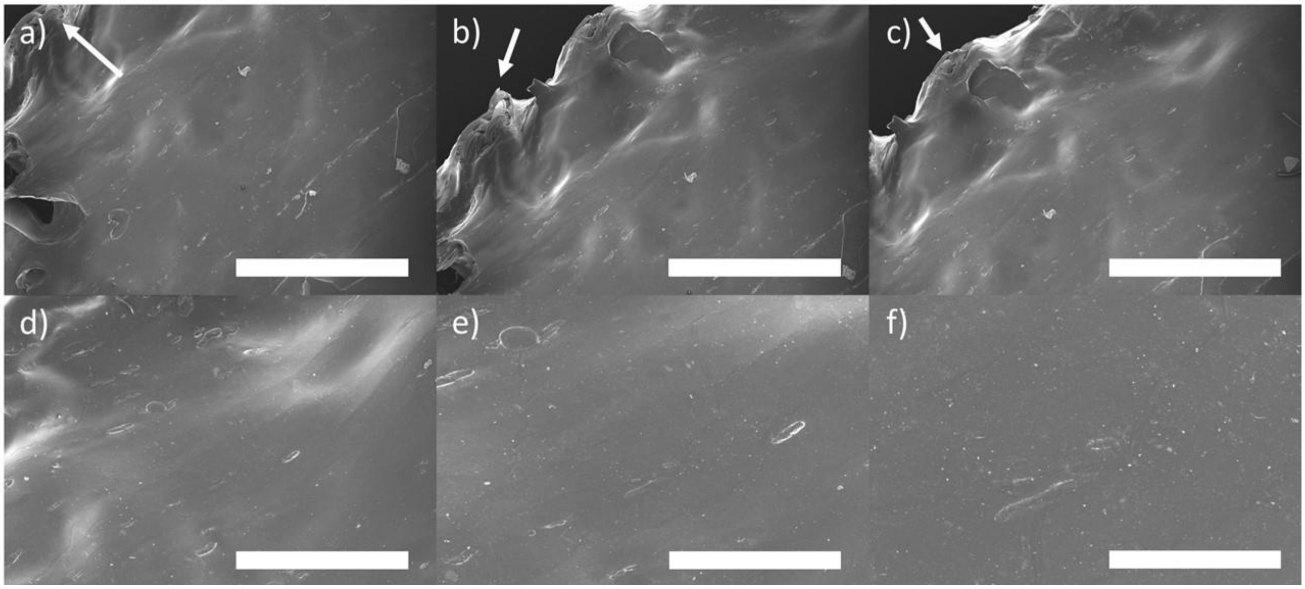

Figure S11. Repetitive mechanical stress-relaxation testing of the EVV. (a-c) Representative SEM images of the EVV after the mechanical stress-relaxation testing. White arrows indicate the place where the sample was cut for preparation with the presence of the PET textile. (d-f) Close-view representative SEM images of the EVV after the mechanical stress-relaxation testing. (a-c) Scale bar = 2 mm. (d) Scale bar = 1 mm. (e) Scale bar = 500 µm. (f) Scale bar = 200 µm.

References

Ackroyd, J. S., Pattison, M., and Browse, N. L. (1985). A study of the mechanical properties of fresh and preserved human femoral vein wall and valve cusps. *J. Br. Surg.* 72, 117–119. doi: 10.1002/bjs.1800720216.

Lu, J., and Huang, H.-Y. S. (2018). Biaxial mechanical behavior of bovine saphenous venous valve leaflets. *J. Mech. Behav. Biomed. Mater.* 77, 594–599. doi: 10.1016/j.jmbbm.2017.10.028.
